# Investigation on the Preparation and Performances of Epoxy-Modified Asphalt Binder and Its Mixtures

**DOI:** 10.3390/ma17112539

**Published:** 2024-05-24

**Authors:** Xiaodong Liu, Zhiheng Wu, Zhaohui Min, Lei Zhang

**Affiliations:** 1 School of Transportation, Southeast University, Nanjing 211189, China; 2 CCCC Highway Consultants Company Limited, Beijing 101300, China

**Keywords:** epoxy-modified asphalt binder, asphalt mixture, pavement performance, tensile strength

## Abstract

Epoxy-modified asphalt binder has been widely used in steel deck pavement due to its excellent properties and it is a potential candidate for long life pavements. However, its short reserve time limits its widespread application in pavement engineering. Therefore, this work developed a novel epoxy-modified asphalt binder composed of a laboratory-made curing agent as a solution. Firstly, optimization of preparation temperature of this new material was studied to balance the requirements of enough construction time and the material strength and elongation. The epoxy-modified asphalt binder, prepared at the optimal temperature of 140 °C, had a reserve time exceeding 120 min, whereas the tensile strength and the elongation at failure were 2.22 MPa and 216%, respectively, which satisfied the standard requirements of paving epoxy material well. Secondly, the asphalt mixture property tests demonstrate excellent high-temperature rutting resistance, water stability and low-temperature anti-cracking ability. Additionally, the compatibility and colloidal stability of this epoxy-modified asphalt binder were analyzed in terms of microphase structure. The uniform microphase distribution of this binder showed by the laser confocal microscope observation in both short-term aging case and long-term aging case, indicates the great compatibility between asphalt and epoxy resin during paving process and service life. Furthermore, fatigue tests were conducted to evaluate the long-term durability. The fatigue life of epoxy-modified asphalt mixtures increased by 435%, 427%, 342%, and 276% under the stress ratios of 0.3, 0.4, 0.5, and 0.6, respectively, compared to those of SBS-modified asphalt mixtures. All these results indicate that the new epoxy-modified asphalt material is promising for applications in pavement engineering, especially suitable for long-life road pavement.

## 1. Introduction

Asphalt binder has long been widely applied in pavement engineering due to its great viscoelastic properties [1,2]. However, with the rapid development of society and the economy, conventionally modified asphalt binders with polymeric modifiers suffer more serious sever environment of increasing traffic volume and vehicle loads. These polymeric modifiers inevitably degrade after being exposed to oxygen, high temperature, and ultraviolet light for a long time, which makes them lack the durability necessary to meet the requirements of current pavement engineering [3,4,5,6,7,8,9]. Therefore, it is necessary to explore better modifiers to solve the inherent structural defects of polymeric modifiers.

Epoxy-modified asphalt binder is a thermosetting pavement material currently used primarily in bridge deck pavement due to its excellent resistance to rutting, deformation, and fatigue [10,11,12,13,14,15]. In 1967, the Adhesive Engineering Company in USA was proactively authorized by Shell Oil to use thermosetting epoxy-modified asphalt binder for the first time in the U.S. for the San Mateo–Hayward Bridge steel bridge deck paving [10]. In 2001, academician Wei Huang et al. used epoxy-modified asphalt binder as a binder to prepare asphalt mixtures, which were successfully used in the deck-paving project of the Nanjing Yangtze River Second Bridge [16]. Since then, thermosetting epoxy-modified asphalt binder has been widely used as a paving material for steel box girder bridges worldwide, achieving better paving results [17,18,19]. Depending on the advantages of thermosetting epoxy-modified asphalt binder, a considerable amount of research work has been carried out on mixture performances in the laboratory to determine its potential engineering applications [20,21,22,23,24,25,26]. Qing Lu et al. [20] developed a bio-based epoxy asphalt binder (BEAB) and found that the formulated BEAB can improve the raveling resistance and stability of porous asphalt mixture without reducing its permeability. Panos Apostolidis et al. [21] investigated the effects of filler type on the durability of epoxy-modified asphalt mixture and found that proportional increase of epoxy in the asphalt mixture led to substantially improved mechanical properties (i.e., strength and toughness). The strength and toughness of mixes containing only EA were higher than all the others, while pure limestone mixes were stronger and tougher than those with hydrated lime. Shuang Shi et al. [25] proposed a new strategy of collaborative toughening to overcome the hot-mix epoxy resin commonly used in the market, which has insufficient toughness, resulting in cracking of steel bridge deck pavement. The results showed that the epoxy asphalt with 15 wt% of toughen agent can decrease the activation energy of epoxy resin from 48.6 KJ/mol to 42.4 KJ/mol. Additionally, the toughening agent can reduce the viscosity and glass transition temperature of epoxy asphalt. Ali Jamshidi et al. [26] comprehensively compared the high-temperature rutting resistance, low temperature cracking resistance and water resistance of traditional SBS-modified asphalt mixtures and different types of thermosetting epoxy-modified asphalt mixtures. The study showed that thermosetting epoxy-modified asphalt binder has excellent road performance and is an ideal material for road paving. In addition, according to previous research, despite the higher cost of epoxy-modified asphalt binder, its paving cost is usually about four times lower than that of conventional polymer-modified asphalt binder from a full life-cycle perspective because epoxy-modified asphalt binder has better fatigue resistance than conventional polymer-modified asphalt binder (such as SBS-modified asphalt binder), which will greatly reduce the repair and maintenance cost in the long-term service period [10,11,27]. Although epoxy-modified asphalt binder is very promising in applications in road pavement, the limited reserve time and long curing period of the main epoxy-modified asphalt binder products can be a challenge in road paving and maintenance. Due to different curing agents, cured epoxy-modified asphalt binder’s curing mechanism and curing law are not the same, so epoxy-modified asphalt binder has a different reserve time and maintenance period. Yong Yan et al. [28] studied the reserve time and maintenance time of epoxy-modified asphalt binder cured by anhydride curing agent at different temperatures, and the results showed that its rotational viscosity is still less than 2 Pa·s after 120 min, which meets the requirements of the reserve time of pavement paving, but the stability of the mixture to reach 40 kN needs more than 30 d. Guoping Feng et al. [29] prepared new amine curing agent, which was stabilized to 77 kN at 60 °C for 4 d of the maintenance, but the reserve time reached 3 Pa·s after only 28 min at a preparation temperature of 140 °C. The long maintenance period of epoxy-modified asphalt binder may affect the progress of the project and make it impossible to open traffic quickly, and the short reserve time limits the spreading of epoxy-modified asphalt binder in road paving as well.

Therefore, in this work, a home-made modified amine was used as a curing agent to develop a road-oriented epoxy-modified asphalt binder with great mechanical properties, short maintenance period, and suitable reserve time. The effect of preparation temperature on the reserve time of epoxy-modified asphalt binder was investigated to determine the optimum preparation temperature. The pavement performance of epoxy-modified asphalt binder and its mixture were comprehensively evaluated and analyzed based on ensuring the reserve time of epoxy-modified asphalt binder. By achieving these objectives, this study provides valuable insights to improve the understanding of epoxy-modified asphalt binder materials and their potential applications in sustainable infrastructure practices.

## 2. Experiment

### 2.1. Raw Materials

#### 2.1.1. Modified Asphalt Binder Materials

The 70# asphalt binder was selected in this study, and its main properties are listed in Table 1. The type of epoxy resin used in this study was E51 obtained from Wuxi epoxy Co., Ltd. (Wuxi, China), and its specification is shown in Table 2. The curing agents used in this study are amine curing agent, which is homemade in the laboratory.

#### 2.1.2. Aggregates

The aggregates used in this work are broken from basalt (Suzhou, China), and the mineral powder is ground from limestone (Suzhou, China). The technical indicators of the coarse aggregate, fine aggregate, and mineral powder are shown in Table 3, Table 4, and Table 5, respectively.

### 2.2. Sample Preparation

#### 2.2.1. Preparation of Epoxy-Modified Asphalt Binder

**Figure 1:**
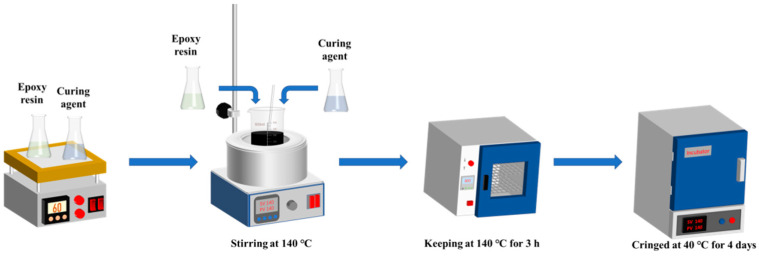
Scheme of preparation process of epoxy-modified asphalt binder.

To investigate the effect of the preparation temperature on the reserve time of the epoxy-modified asphalt binder, the epoxy-modified asphalt binder was produced by electromagnetic stirrer at 100 °C, 120 °C, 140 °C, and 160 °C, respectively, in which the mass ratio of curing agent:epoxy resin:matrix asphalt was 1:1.4:4. The preparation process was shown in Figure 1. Firstly, the mixture of curing agent and epoxy resin was stirred at 140 °C to obtain a reactive pre-crosslinker. Then, the basic asphalt binder was added to the reactive pre-crosslinker. Finally, they were stirred fully at 140 °C for 2 h and cringed at 40 °C for 4 days to form epoxy-modified asphalt binder.

#### 2.2.2. Aging Treatment of Epoxy-Modified Asphalt Binders

To investigate the aging resistance and modification mechanism of epoxy-modified asphalt binder, three basic asphalt binders with different aging degrees were used to prepare epoxy-modified asphalt binder. The short-term aged basic asphalt binders were prepared by the rolling thin film oven test (RTFOT, SYD-0601, Shanghai Changji Geological Instrument Co., Ltd., Shanghai, China) according to the standard ASTM D2872, in which 35 g of molten modified asphalt binder was placed into the glass bottle (D4 cm × H15 cm) and then was heated at 163 °C for 85 min. The long-term aging was conducted on the pressure aging vessel (PAV, PAV-1 aging system, Shanghai Changji Geological Instrument Co., Ltd., Shanghai, China) using the procedure of ASTM D6521, in which 50.0 g of modified asphalt binder treated by RTFOT was added into the stainless steel dishes (D140 mm × H15 mm) to form a film with a thickness of about 3.2 mm and then was exposed into the air with 2.1 MPa ± 0.1 MPa at 90 °C for 20 h. The ultra-long-term aging asphalt binder was treated the same way as long-term basic asphalt binder, with an aging time of 40 h. Then, the aging epoxy-modified asphalt binder was prepared following Section 2.2.1.

#### 2.2.3. Preparation of Epoxy-Modified Asphalt Mixtures

**Figure 2:**
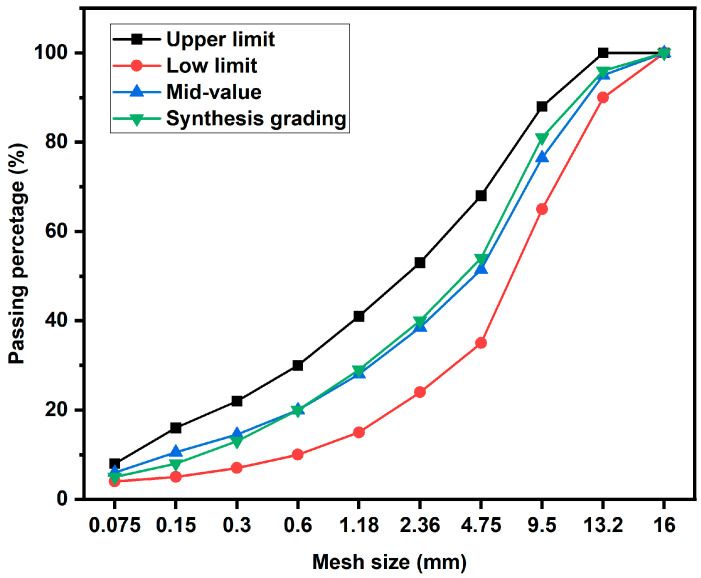
Gradation curves of epoxy-modified asphalt mixtures.

The predetermined asphalt ratio (oil/stone ratio) of the epoxy-modified asphalt mixture was 6.5%. The optimum asphalt ratio in this paper is consistent with the research in literature which adopted an optimum asphalt ratio of 6–7% [22,31]. The epoxy-modified asphalt binder raw binder was prepared following the method outlined in Section 2.2.1. Subsequently, the epoxy-modified asphalt binder was mixed with aggregate at 140 °C, and immediately put into Marshall molding machine to prepare Marshall specimens, in which the temperature of the mixtures was not strictly controlled. The curing and conditioning steps for the Marshall specimens of epoxy-modified asphalt binder were conducted in alignment with the binder preparation process. The AC-13 aggregate gradation, in which the maximum nominal size is 13 mm, is selected to preparation the epoxy-modified asphalt binder mixture according to the requirements of the standard (Technical Specification for Construction of Highway Asphalt Pavement, JTGF40-2004) in China [32], and it is listed in Figure 2.

### 2.3. Temperature Optimization for the Binder Preparation

#### 2.3.1. Rotational Viscosity (RV) for Workability Evaluation

The viscous behavior of the epoxy-modified asphalt binder was measured by a Changji NDJ-1C Brookfield rotational viscometer (Shanghai, China) [33]. The test was performed at 100 °C, 120 °C, 140 °C, and 160 °C with the spindle set to 27 respectively.

#### 2.3.2. Tensile Test for Rating the Tensile Strength and the Elongation

The tensile strength and elongation at the break of the epoxy-modified asphalt binder were measured by the CMT5105 electronic universal tensile testing machine at room temperature and a rate of crosshead movement of 500 mm/min, in which the measured sample in this test was cut into dumbbell-shaped samples according to ASTM D638, and a minimum of five measurements were made repeatedly for each group of samples [34].

#### 2.3.3. Marshall Stability Test

The Marshall Stability test was performed in accordance with the standard (JTG E20-2011 and ASTM D6927) [31]. Based on standard, the specimens were prepared with the specified temperature by immersing in a water bath at a temperature of 60 °C ± 1 °C for a period of 45 min and loaded at a constant rate of deformation of 50.8 mm/min until the maximum load was reached. These measurements were repeated at least three times to obtain an average value.

### 2.4. Anti-Aging Performance and Microscopic Mechanism of Epoxy-Modified Asphalt Binder

A Leica TCS SPE confocal microscope (CLSM) was used to characterize the microstructure images of the epoxy-modified asphalt binder. The CLSM images were obtained from the focal plane with point-by-point laser scanning, which can symbolize the cross-section microstructure from the focal plane of the specimen because any noise resulting from out of focus plane will be removed optically. This technique can more authentically represent the microstructure of the materials compared with conventional widefield microscopy.

### 2.5. Pavement Performance Investigation of Epoxy-Modified Asphalt Mixture

The high-temperature stability, low-temperature cracking resistance, and water stability of this new epoxy-modified asphalt mixture were completely investigated using the rutting test, the three-point bending beam test, and the Marshall immersion test, respectively.

#### 2.5.1. Rutting Test

The rutting test was conducted in accordance with JTG E20-2011 (basically the same as AASHTO T324-14) [30]. Slab specimens with dimensions of 300 mm × 300 mm × 50 mm were produced using a roller compactor, and were then put into a thermostat at 60 °C to maintain a constant temperature and dry environment. Wheel-tracking tests were performed using a wheel-tracking test system with a solid rubber wheel at a speed of 42 passes per minute and a pressure of 0.7 MPa. The rutting depth was recorded by a data collector every 20 s, and the dynamic stability was calculated from the deformation curves. Three replicates were used for each test, and the final data were averaged for each test.

#### 2.5.2. Three-Point Bending Beam Test

The three-point bending beam test was conducted in strict accordance with test method T 0728-2011 in JTG E20-2011 and RILEM TC 50-FMC [30]. The test beam specimen had dimensions of 250 mm length, 30 mm width, and 35 mm height, identical to the plate specimen used in the wheel track test. The specimens were prepared and placed in a constant temperature bath at −10 °C. They were then loaded onto a creep tester at a rate of 50 mm/min, and the load and deflection at the center of the beam specimen were recorded [35]. Each test was replicated three times, and the final data were averaged.

#### 2.5.3. Marshall Immersion Test

The water stability of epoxy-modified asphalt mixture was assessed using the Marshall immersion test, as outlined in T0709-2011 JTG E20-2011 and ASTM D6927. Marshall specimens were compacted with 75 blows per side and then pretreated in a water bath at 60 °C for 48 h. The specimens were loaded at a rate of 50.8 mm/min until destruction, and the stability of the immersed homes was recorded by a data acquisition device [36]. Each test was conducted with five replicates, and the final data were averaged.

#### 2.5.4. Fatigue Test

The specimen beams used in the three-point bending fatigue test were cut from rut board, featuring a size of 250 mm × 30 mm × 35 mm. The three-point bending fatigue test was conducted with stress control mode under 15 °C. The specimen beams were loaded with a half-sine wave with a wave width of 200 ms, and the loading frequency was 10 Hz.

## 3. Results and Discussion

### 3.1. Reserve Time of Epoxy-Modified Asphalt Binder

**Figure 3:**
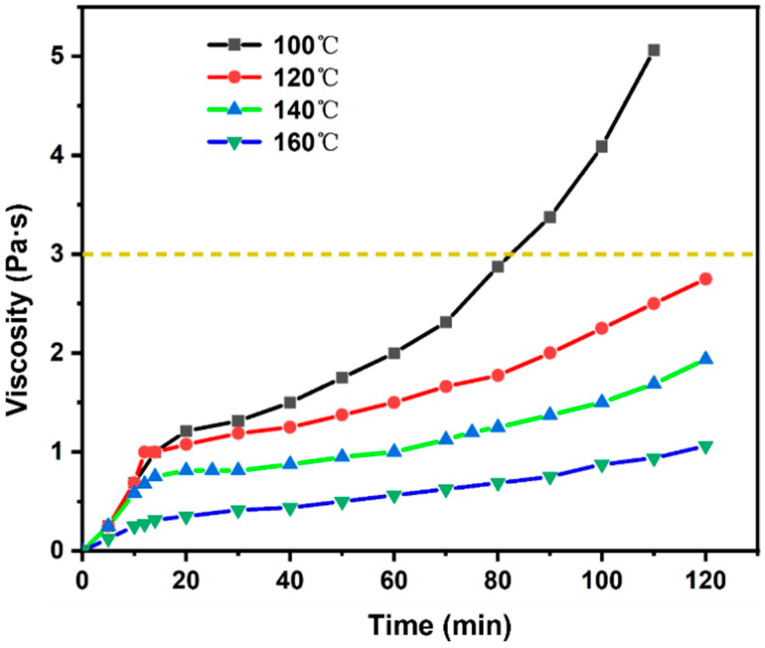
Viscosity–time curves of epoxy-modified asphalt binder at different temperatures.

To investigate the reserve time of the new epoxy-modified asphalt binder, the viscosity growth curves of the unaged epoxy-modified asphalt binder were measured at different temperatures. According to the standard of AASHTO T312, the viscosity of modified asphalt binder is required within 3 Pa·s to meet the paving of modified asphalt binder. Since the viscosity of epoxy-modified asphalt binder increases with curing time, the time when the viscosity of epoxy-modified asphalt binder reaches to satisfy 3 Pa·s is defined as the reserve time, which is generally not less than 120 min [27]. As Figure 3 shows, different preparation temperatures have a significant effect on the viscosity growth of epoxy-modified asphalt binder within 2 h. Epoxy asphalt binder contains the behaviors of chemical cross-linking, so its viscosity increases continuously with the increase of reaction time. The viscosity growth curves of epoxy-modified asphalt binder in Figure 3 show that when the preparation temperature is 100 °C, the viscosity of epoxy-modified asphalt binder grows faster, and its viscosity has already exceeded 3 Pa·s at 80 min. Therefore, considering the requirements of the principle of the reserve time, the preparation temperature of 100 °C does not comply with the restrictive requirements of the viscosity growth of not more than 3 Pa·s in 120 min. Except for the preparation temperature of 100 °C, all other preparation temperatures satisfy the requirement of the reserve time of epoxy-modified asphalt binder. In addition, the higher the preparation temperature, the slower the viscosity growth. This phenomenon may be due to the curing agent properties of this material. Since the viscosity of this material grows relatively slowly in the early stage, the higher the mixing temperature in the early stage, the lower the viscosity of the asphalt binder, and the base of the viscosity growth is small, which leads to a smaller viscosity growth. The viscosity–time curves of epoxy-modified asphalt binder show that it is very suitable for the paving requirements of current pavement engineering.

### 3.2. Mechanic Performance of Epoxy-Modified Asphalt Binder

**Figure 4:**
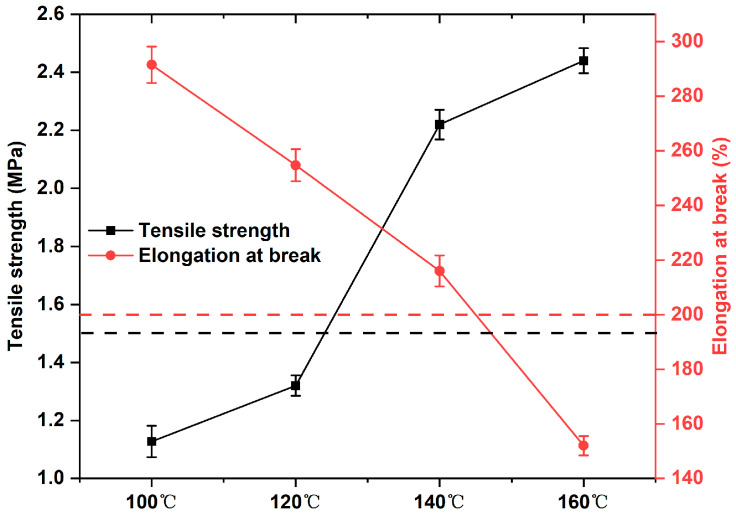
Tensile results of epoxy-modified asphalt binder at different temperatures.

The preparation and curing temperature of epoxy-modified asphalt binder has a large impact on the performance of modified asphalt binder. Thus, the reserve time of the modified asphalt binder met the requirements, and a tensile test was used to further investigate the effect of preparation temperature on the mechanical properties of the unaged epoxy-modified asphalt binder. According to the standard (GB/T 30598-2014 and ASTM D7369), the tensile strength and elongation at break of epoxy-modified asphalt binder should be greater than 200% and 1.5 MPa, respectively. As Figure 4 shows, with the increase in preparation temperature, the higher the tensile strength of the epoxy-modified asphalt binder material are, the lower the elongation at break of the epoxy-modified asphalt binder material are. This is because as the mixing temperature increases, the curing reaction in 2 h (120 min) is more adequate, and the base of strength growth in the post-conditioning process is increased so that a higher post-strength will be obtained. Meanwhile, Figure 4 shows that only at 140 °C, the tensile strength and elongation can meet the requirement that the tensile strength and elongation at break of modified bitumen should be greater than 200% and 1.5 MPa, respectively.

### 3.3. Marshall Stability Test of Epoxy-Modified Asphalt Mixture

**Figure 5:**
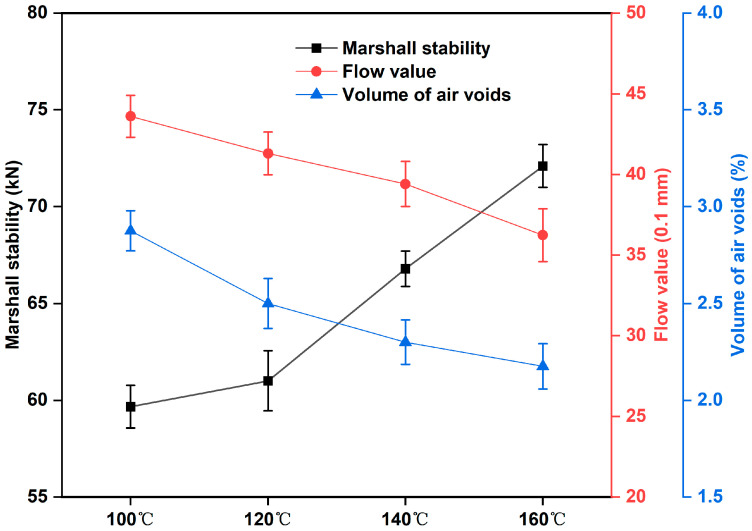
Marshall stability test of epoxy-modified asphalt mixture at different temperatures.

The Marshall stability test was utilized to assess the rutting resistance of asphalt mixtures. The Marshall stability (MS) and flow value (FV) were the peak load and corresponding vertical deformation in the Marshall test, respectively. Figure 5 exhibits that different preparation temperatures have a significant impact on the mechanic strength of epoxy-modified asphalt mixtures, in which the higher the preparation temperature, the greater the MS of the corresponding epoxy asphalt mixtures and the smaller the FV of epoxy-modified asphalt mixtures. The results of the Marshall stability test on epoxy-modified asphalt binder are consistent with the changing law of tensile strength and elongation at break of epoxy-modified asphalt binder. In addition, the MS values and FL values of all epoxy-modified asphalt mixtures under the four preparation temperatures can meet the requirements of the corresponding indexes in the standard of GB/T 30598-2014 and ASTM D6927, in which the Marshall stability is greater than 40 kN, and the flow value is between 20–50. As we know, the higher the preparation temperature of modified asphalt binder, the more its production cost will also increase, but the preparation temperature is lower than 120 °C, which will also enhance the mixing plant temperature control difficulties. The above tests show that the epoxy-modified asphalt binder prepared at 140 °C can fully meet the performance requirements of epoxy-modified asphalt binder for road engineering in terms of reserve time and mechanical properties.

### 3.4. Confocal Laser Scanning Microscopy Analyses

**Figure 6:**
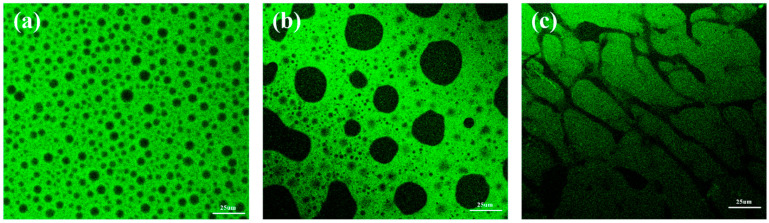
Confocal laser scanning microscopy images of epoxy-modified asphalt binder with different aging basic asphalt binder: (**a**) short-term aging basic asphalt binder, (**b**) long-term aging basic asphalt binder, (**c**) ultra-long-term aging basic asphalt binder.

The aging of epoxy-modified asphalt binder is mainly due to the ageing of the basic asphalt binder [31,37]. To investigate the effect of the ageing basic asphalt binder on epoxy modified asphalt binder, the micro-morphological state of the epoxy resin in epoxy-modified asphalt binder was observed using a laser confocal microscope with an excitation wavelength of 488 nm. As shown in Figure 6, the absence of fluorescent substances within the asphalt binder does not emit excitation light and is shown as a black image, while the epoxy resin is irradiated by the laser to emit excitation light and is shown as a green image. Figure 6 shows that the green area, which represents the epoxy resin, has a much larger area than the black area representing the asphalt binder. This suggests that the epoxy resin and asphalt binder have formed a spatial three-dimensional mesh structure. As shown in Figure 6a, the asphalt binder exhibits black dots in a uniform distribution of the epoxy system after short-term aging, which indicates that the new epoxy-modified asphalt binder has great compatibility and colloidal stability. After long-term aging, the basic asphalt binder aggregates into a black island structure distributed in the epoxy resin phase, and the area and size of the black area increase significantly. After ultra-long-term aging, the phenomenon of basic asphalt binder aggregation becomes more pronounced, forming a structure like a neural network with connected island-like protruding vesicles. It appears that the compatibility between the asphalt binder and the epoxy resin deteriorates as the asphalt binder ages.

Furthermore, the thermosetting time and polymerization process of the asphalt-mineral mixture at high temperature may significantly influence its compatibility and strength properties, which should be studied in further research.

### 3.5. Technical Properties of Epoxy-Modified Asphalt Mixture

Epoxy-modified asphalt mixtures were prepared using epoxy-modified asphalt binder prepared at 140 °C. The technical requirements of epoxy asphalt mixture were used to evaluate the pavement performance of the mixture, and the test results are shown in Table 6. According to the CJJ/T 279-2018 and AASHTO T324-14, the specification requires the dynamic stability (DS) value of epoxy-modified asphalt mixture to be not less than 5 × 10^3^ times/mm, and the maximum flexural strain (ε) value and the residual stability (RS) value of them are required to be not less than 2.5 × 10^−3^ and 85%, respectively.

The rutting test results in Table 6 show that the DS value of this new epoxy-modified asphalt mixture meets the technical specifications required in the standard (CJJ/T 279-2018 and AASHTO T324-14) and is almost four times better than the standard. At the same time, the rut depth grade of this new epoxy-modified asphalt mixture is 0.6 mm. Therefore, the rutting test results show that this new epoxy-modified asphalt mixture has excellent high-temperature rutting resistance. Meanwhile, from the results of the three-point bending beam test, the *ε* value of this new epoxy-modified asphalt mixture is 3.9 × 10^−3^, which is greater than the technical requirements of the standard (CJJ/T 279-2018 and RILEM TC 50-FMC), meaning that this new epoxy-modified asphalt binder mix has better low-temperature cracking resistance. In addition, the results of the Marshall immersion test shows that the R_S_ of this new epoxy-modified asphalt mixture is 93.6, which is significantly greater than the 85% required in the standard (CJJ/T 279-2018 and AASHTO T324-14), proving that this new epoxy-modified asphalt binder has great water stability. Based on the above test results, this new epoxy-modified asphalt mixture has great technical properties and can completely meet the requirements of the epoxy-modified asphalt binder in the standard (CJJ/T 279-2018).

**Figure 7:**
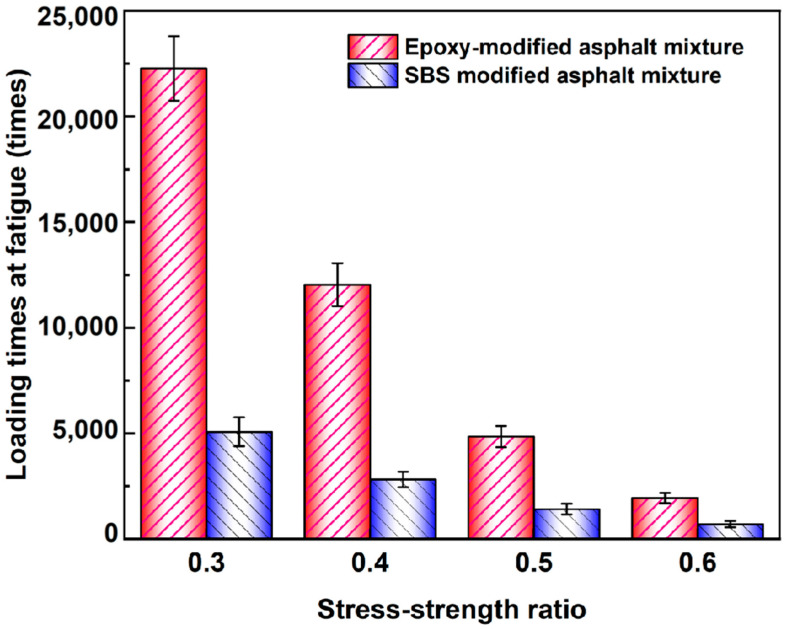
The fatigue life of epoxy-modified asphalt and SBS-modified asphalt mixture under different stress–strength ratios.

To evaluate the fatigue resistance of epoxy-modified asphalt binder, the fatigue life of the new epoxy-modified asphalt mixture and the traditional SBS-modified asphalt mixture were measured by three-point bending fatigue test. The number of loading times corresponding to the fracture of the specimen beams in the three-point bending fatigue test is defined as the fatigue life, the larger the fatigue life, the better the fatigue resistance of asphalt mixtures. As can be seen from Figure 7, the fatigue life of epoxy-modified asphalt mixtures is higher than that of SBS-modified asphalt mixtures under all the same stress-strength ratio conditions. The fatigue life of epoxy-modified asphalt mixtures increased by 435%, 427%, 342%, and 276% under stress ratios of 0.3, 0.4, 0.5, and 0.6, respectively, compared to those of SBS-modified asphalt mixtures, which indicates that this new epoxy-modified asphalt mixture has excellent fatigue performance compared with ordinary asphalt mixtures.

## 4. Conclusions

In this work, a novel epoxy-modified asphalt binder composed of a laboratory-made curing agent was developed. The optimum preparation temperature of this new material was studied to balance the requirements of enough construction time and the material strength and elongation. Then the mixture performances were investigated, including a rutting test, a three-point bending beam test, and a Marshall immersion test. Furthermore, long-term durability for the pavement using epoxy-modified asphalt mixture was comprehensively evaluated, for which the compatibility and colloidal stability of this epoxy-modified asphalt binder were analyzed in terms of microphase structure, and the fatigue tests of three-point bending beam were conducted under different stress ratios. Based on the research results, the following conclusions can be drawn:

Based on the viscosity curve and the tensile test of the new epoxy-modified asphalt binder, its optimum preparation temperature is approximately 140 °C. Its reserve time is much longer than 120 min, and its tensile strength and elongation at failure are 2.22 MPa and 216%, respectively, which is quite enough to satisfy the construction time and pavement performance.

The dynamic stability (DS) value of the new epoxy-modified asphalt mixture in the rutting test is 22,000 times/mm, and the residual stability (RS) value in the Marshall immersion test is 93.6%. From the three-point bending beam test, the maximum flexural strain value is 3.9 × 10^−3^. These results indicate that the new epoxy-modified asphalt mixture has outstanding high-temperature rutting resistance, water stability, and low-temperature anti-cracking ability, which provide it with good application prospects in pavement engineering projects.

In addition, the uniform microphase structure of the new epoxy-modified asphalt binder based on the laser confocal microscope observation in both short-term aging case and long-term aging case, indicates the great compatibility between asphalt and epoxy resin during paving process and service life.

Furthermore, the fatigue life of epoxy-modified asphalt mixtures increased by 435%, 427%, 342%, and 276% under stress ratios of 0.3, 0.4, 0.5, and 0.6, respectively, compared to those of SBS-modified asphalt mixtures, which indicates that this new epoxy-modified asphalt mixture has excellent fatigue resistance.

In summary, this new epoxy-modified asphalt binder and mixture are promising for application in pavement engineering, especially in long-life road pavement.

## Figures and Tables

**Table 1:** Properties of base asphalt binder.

Items	Technical Indexes	Results	Standard
Penetration (25 °C, 100 g, 5 s)/0.1 mm	60–80	69	JTG E20-T 0604 [30]
Softening point (°C)	≥46	47.60	JTG E20-T 0606 [30]
60 °C dynamic viscosity (Pa·s)	≥180	256	JTG E20-T 0620 [30]
15 °C ductility (15 °C, 5 cm/min)/cm	≥100	165	JTG E20-T 0605 [30]

**Table 2:** Properties of epoxy resin.

Items	Technical Indexes	Results	Standard
Viscosity (Pa·s)	11–15	14.66	ASTM D1652
Epoxy equivalent (g/ep)	185~192	189	ASTM D1084

**Table 3:** The technical indicators of coarse aggregate.

Items	Results
Crushing value (%)	9–13
Los Angeles abrasion loss (%)	17.1
Water absorption (%)	0.6
Apparent relative density (g/cm^3^)	2.796
<0.075 mm content (%)	0.7

**Table 4:** The technical indicators of fine aggregate.

Items	Results
Apparent relative density (g/cm^3^)	2.644
Angularity	42.9
Sturdiness (%)	5–13
Mud content (%), ≤	2.644

**Table 5:** The technical indicators of mineral powder.

Items	Results
Apparent relative density (g/cm^3^)	2.770
Water absorption (%)	0.45
Hydrophilic coefficient (%)	0.62
Plasticity index (%)	2.56

**Table 6:** Technical properties of epoxy-modified asphalt mixture.

Rutting Test		Three-Point Bending Beam Test		Immersion Marshall
Rut Depth (mm)	*DS* (10^3^ Cycle/mm)	*σ0* (MPa)	ε (10^−3^)	*R_S_* (%)
0.6	22,000	30.58	3.9	93.6

## Data Availability

The original contributions presented in the study are included in the article, further inquiries can be directed to the corresponding author.
